# Myélome multiple et autogreffe des cellules souches hématopoïétiques sans cryoconservation: expérience du Service d´Hématologie Clinique de Casablanca au Maroc

**DOI:** 10.11604/pamj.2021.39.105.18994

**Published:** 2021-06-04

**Authors:** Salma Fares, Halima Hadri, Mohamed Rachid, Tarik Moutiqui, Bouchra Oukkache, Asmaa Quessar

**Affiliations:** 1 Service d´Hématologie et d´Oncologie Pédiatrique, Hôpital 20 Août 1953, CHU Ibn Rochd, Casablanca, Maroc,; 2 Service d´Hémato-biologie, CHU Ibn Rochd, Casablanca, Maroc

**Keywords:** Myélome multiple, autogreffe, cellules souches hématopoïétiques, sans cryoconservation, Multiple myeloma, autologous, haematopoietic stem-cell transplantation, without cryopreservation

## Abstract

La chimiothérapie à haute dose suivie d´autogreffe des cellules souches hématopoïétiques (ASCT) reste le traitement de choix du myélome multiple chez les sujets âgés ≤ 65 ans. Dans les pays en développement, l´ASCT sans cryoconservation, permet de réduire les coûts d´hospitalisation et des installations nécessaires. Pour évaluer cette procédure, une étude prospective, longitudinale et ouverte a été conduite au Service d´Hématologie Clinique de Casablanca au Maroc. Sur 24 mois, 64 patients ont été colligés. Après le traitement d´induction, la réponse globale (Rémission complète + Très bonne réponse partielle) était à 67,2% (43 patients). La richesse moyenne du greffon autologue était de 12.97x10^6^ CD34+/Kg [2.4- 53x10^6^ CD34+/Kg] et la durée médiane d´hospitalisation était de 20,5 jours [14-60 jours]. La réponse globale en post autogreffe était de 84% (54 patients). A 24 mois, l´estimation de la survie globale (OS) était à 83,5%, la médiane d´OS n´était pas atteinte et la survie sans progression (PFS) à 65,9% avec une médiane de la PFS à 24,1 mois avec un intervalle de confiance à 95% [21,7-26,5mois]. L´ASCT sans cryoconservation reste une excellente alternative dans notre contexte, ce qui permet de réduire les délais d´attente, et les coûts de congélation.

## Abstract (en)

High-dose chemotherapy followed by autologous hematopoietic stem cell transplantation (AHSCT) is the gold standard treatment for multiple myeloma in subjects aged ≤ 65 years. In developing countries, AHSCT without cryopreservation reduces the costs of hospitalization and all necessary equipments. We conducted a longitudinal, prospective, open study to evaluate this procedure at the Department of Clinical Hematology, Casablanca, Morocco. Data from the medical records of 64 patients were collected over a period of 24 months. After induction therapy, the overall response (complete remission + very good partial response) was 67.2% (43 patients). The mean CD34-cell count in the autograft was 12.97*10^6^ /kg [2.4- 53*10^6^ Cd34+/kg] and the median length of hospitalization was 20.5 days [14-60 days]. The overall response after autograf was 84% (54 patients). At 24 months follow-up, overall survival (OS) was 83.5%, median OS was not reached and progression-free survival (PFS) was 65.9%, with a median PFS of 24.1 months with 95% confidence interval [21.7-26.5 months]. Autologous hematopoietic stem cell transplantation without cryopreservation is an excellent alternative in our context reducing wait times and freezing costs.

## Introduction

Le myélome multiple (MM) représente environ 1% de l´ensemble des cancers et 13% des hémopathies malignes. Dans les pays occidentaux, l´incidence annuelle ajustée à l´âge est de 5.6 cas pour 100000 habitants [1]. Au Maroc, l´incidence annuelle estimée du MM est de 1 cas pour 100000 habitants selon le Registre des Cancers de la Région Grand Casablanca de 2004 [2].

La chimiothérapie à haute dose suivie d´autogreffe des cellules souches hématopoïétiques (ASCT) ont été développées dans les années 1980 et elles sont considérées depuis, comme le traitement standard de première ligne chez les patients atteints de MM et éligibles à l´autogreffe depuis les années 1990. L´ASCT réalisée après l´utilisation d´une combinaison des nouveaux agents en chimiothérapie d´induction permet d´obtenir une réponse profonde traduisant une survie sans progression (PFS) plus longue et surtout une amélioration de la survie globale (OS) [3,4]. Toutefois, l´ASCT est une ligne thérapeutique couteuse qui nécessite beaucoup de ressources, telle que la congélation des cellules souches hématopoïétiques recueillies afin de maintenir leur viabilité jusqu´à la réinjection du greffon. Le stockage des cellules souches prélevées dans des réfrigérateurs classiques à +4°c est une alternative de cryoconservation.

En 2007, Wannesson *et al*. ont publié une revue systématique dans laquelle ils ont démontré la faisabilité et la sécurité des ASCT sans cryoconservation du greffon aussi longtemps que nous utilisons des protocoles de conditionnements courts [5]. L´ASCT sans cryoconservation est utilisée par certaines institutions dans des régions à ressources économiques limitées tout en ayant une infrastructure adéquate pour traiter les hémopathies malignes [6,7].

Au Maroc, l´activité d´ASCT avec cryoconservation pour les hémopathies malignes y compris le MM a été démarrée depuis juillet 2004. Vu le nombre de patients et l´importance de l´ASCT dans le traitement du MM en 1^ère^ ligne et puisque les patients étaient mis sur une liste d´attente nationale avec des délais de prélèvement des cellules souches et de congélation qui pouvaient dépasser un an, la nécessité de créer une activité ASCT sans cryoconservation s´avérait primordiale. Pour cela, le Service d´Hématologie Clinique de Casablanca (SHOP) a lancé une activité d´ASCT sans cryoconservation pour le traitement du MM à partir de janvier 2014 et une étude prospective simultanée a été menée pour évaluer la faisabilité et l´efficacité de cette procédure et rapporter les résultats préliminaires concernant l´évolution et les taux de réponse.

## Méthodes

Une étude prospective longitudinale et ouverte à but descriptif sur 24 mois a été menée au Service d´Hématologie Clinique de Casablanca. Le recrutement des patients a été démarré en janvier 2014 et il a été clôturé en décembre 2015 avec une analyse des données en juin 2016.

**Critères d´inclusion:** patients âgés ≤ 65 ans; MM de novo retenu selon les critères de l´International Myeloma World group (IMWG) [8] et stratifiés en 3 sous-groupes selon l´International Staging System (ISS) [9] et selon l´étude cytogénétique réalisée à la recherche d´une translocation (4, 14), une délétion du 13 ou du 17p qui ont un pronostic défavorable avec une survie médiane diminuée; patients en rémission complète (RC), en très bonne réponse partielle (TBRP) ou en réponse partielle (RP) selon les critères de l´IMWG [10] après un traitement d´induction.

Les patients ont reçu comme chimiothérapie d´induction soit CTD (Cyclophosphamide: 500 mg J1, J8 et J15; Dexaméthasone: 40 mg/j per os ou injectable de J1 à J4 et de J12 à J15; Thalidomide: 100 à 200 mg/j per os le soir en continu) ou VTD (Bortézomib: 1,3 mg/m^2^ en intraveineux J1, J4, J8 et J11; Dexaméthasone: 40 mg/j per os ou injectable de J1 à J4 et de J12 à J15; Thalidomide: 100 à 200 mg/j per os le soir en continu).

### Procédure d´intensification

Le greffon a concerné exclusivement des cellules souches périphériques (CSP), mobilisées par du G-CSF (Lenograstim) pendant 4 jours à raison de 10μg/kg/j ou 5 jours si le comptage des cellules CD34+ sur sang périphérique n´était pas suffisant après 4 jours de mobilisation par le G-CSF, par la suite, le recueil des CSP a été fait par leucaphérèse à l´aide d´un appareil d´aphérèse type Cobe Spectra ou Optia. Le comptage des cellules CD34+ a été fait par cytométrie en flux sur sang périphérique et sur le greffon et le prélèvement a été refait le jour suivant si la richesse du greffon en cellules CD34+ < 2,10μ/Kg. La conservation a été faite à l´état liquide à +4°c pendant 48-72 heures.

Le conditionnement myéloablatif a été fait à base de Melphalan 200mg/m^2^ à J-1. La réinjection des CSP a été faite à J0 par voie veineuse périphérique sous monitorage, après vérification de la poche, de l´identité du patient, et après vérification ultime de la compatibilité au lit du patient. L´aplasie a été gérée en respectant les règles d´isolement et de protection. G-CSF (Lenograstim) a été administré à partir du 5ème jour de la réinjection à raison de 5μg/kg/j et est continué jusqu´à un taux de PNN > 0,5Giga/L pendant 72 heures consécutives. La neutropénie fébrile a été gérée selon les recommandations de l´IDSA (Infectious Diseases Society of America) de 2010 adaptées à l´écologie et aux habitudes du service.

Les produits sanguins labiles reçus étaient phénotypés filtrés et irradiés avec comme seuil de transfusion: taux d´Hb ≤ 8g/dl ou < 10g/dl si l´anémie mal tolérée ou comorbidités associées; taux de plaquettes < 10G/L ou >10G/L avec une neutropénie fébrile ou un syndrome hémorragique actif. Le patient était déclaré sortant du côté greffe s´il était apyrétique pendant > 48h-72h sans signes d´infections décelables, avec un taux de PNN≥1GL, un taux d´HB ≥8g/dl, un taux de plaquettes ≥ 20G/L et une indépendance aux transfusions plaquettaires > 48h. L´évaluation de la réponse en post-ASCT a été faite à J100 post réinjection des CSP par un examen physique complet, un bilan biologique électrophorétique et un myélogramme. Deux cures de consolidation par CTD ou VTD ont été proposées à partir du J60 post-ASCT.

### Analyse statistique des données

Les données ont été recueillies en utilisant une fiche d´exploitation et traitées par logiciel SPSS18.0. Les variables quantitatives de distribution gaussiennes ont été décrite en moyenne et les variables quantitatives de distribution non gaussiennes ont été décrites en médiane et intervalle interquartiles. Les variables qualitatives ont été décrites en effectifs et pourcentage. Les courbes de survie globale (OS) et de la survie sans progression (PFS) ont été calculées selon la méthode de Kaplan- Meier et toutes les estimations d´OS et de PFS ont été accompagnées par leur erreur standard (ES). Les médianes de durée d´OS et de PFS ont été calculées en utilisant un intervalle de confiance à 95%.

La mortalité liée au traitement (TRM), a été définie par la survenue d´un décès due à une cause en dehors de la progression ou la rechute du MM durant les 100 premiers jours suivant la réinjection des CSP. La durée médiane du suivi depuis la date du diagnostic était de 25 mois [7-79mois] et les données ont été censurées le 30 juin 2016.

### Considérations éthiques

Tous les patients ont signé un consentement éclairé avant l´hospitalisation au côté greffe; dans lequel ils déclarent que toute la procédure d´ASCT, ainsi que les complications ou les effets secondaires ont été bien expliqués par le médecin responsable du côté greffe et qu´ils acceptent les faits et les conséquences. L´anonymat et la confidentialité ont été respecté dans toutes les étapes de traitement des données.

## Résultats

### Caractéristiques de la population d´étude

Sur une période de 24 mois, l´étude a colligé 64 patients, dont 45,3% (29/64) en 2014 et 54,7% (35/64) en 2015. Le nombre moyen de patients autogreffés par mois était de 2,66 patients [0-5] et la durée médiane du séjour était de 20,5 jours [14-60 jours]. L´âge médian de la population était de 57 ans [30-65 ans], dont 21,8% (14/64) étaient âgés de moins de 50 ans et le sexe ratio H/F était de 1,75. Les patients colligés ont été répartis selon le type du MM, classés selon la classification de Durie et Salmon dans 98,4% et stratifiés selon l´ISS dans 75%. L´étude cytogénétique a été réalisée chez 12 patients soit 18,7% objectivant la t(11 ,14) chez un patient, t(4,14) et la délétion 17p chez deux patients, la délétion 13q chez un patient, et un caryotype normal chez quatre patients. Le traitement d´induction était fait essentiellement de cures CTD chez 51 patients (76,5%) et la réponse globale (RC+TBRP) après le traitement d´induction a atteint 67,2% (Tableau 1).

**Tableau 1:** répartition des patients en fonction du type de myélome multiple, l´International Staging System et la réponse thérapeutique

	N (64)	%
**MM à chaines légères**	25	39
Kappa	13	52
Lambda	12	48
**MM à IgG**	26	40.6
**MM à IgA**	8	12.5
**MM sans typage électrophorétique**	5	7.9
**Classification de Durie et Salmon**	63	98.4
**Stade I**	2	3.2
**Stade II**	4	6.3
**Stade III**	57	90.5
Stade A	50	79.4
Stade B	13	20.6
**Score Pronostique international (ISS)**	48	75
I	4	8.3
II	12	25
III	32	66.7
**Statut avant ASCT**		
Rémission complète	2	3.2
Très bonne réponse partielle	41	64
Réponse partielle	21	32.8
**Nombre de lignes thérapeutiques avant ASCT**		
1	56	87.5
2	8	12.5

MM: myélome multiple, ASCT: autogreffe des cellules souches hématopoïétiques

### Procédure d´ASCT sans cryoconservation et complications

La durée de mobilisation par le G-CSF était de 4 jours chez 52 patients soit 81,2% et de 5 jours chez 12 patients soit 18,8%. Le comptage des cellules CD 34+ sur sang périphérique a été fait chez 82,8% (53/64) patients. Les CSP ont été recueillies après une seule séance de leucaphérèse dans 95,3% (61/64) des cas et après 2 séances dans 4,7% soit 3 cas et la richesse moyenne du greffon autologue était de 12,97x10^6^ CD34+/Kg [2.4-53x10^6^ CD34+/Kg]. Aucun patient n´a présenté un incident allergique au moment de la réinjection des CSP ou un échec de réinjection du greffon nécessitant une 2^e^réinjection des CSP. La durée médiane d´aplasie était de 9 jours [6-30 jours] avec une durée médiane de recouvrement de: Neutrophiles à 13 jours [9-37 jours] en comptant 72 heures consécutifs avec un taux de PNN ≥ 0,5G/L et les plaquettes > 20G/L à 11jours [9-32 jours]. Tous les patients ont eu un besoin transfusionnel en unités plaquettaires (UP) et/ou en concentrés globulaires (CG) avec une médiane de transfusion en UP de 13 [4-82 UP] et une médiane de transfusion en CG de 2 [0-8 CG]. Les diarrhées motrices étaient présentes chez 25 patients (39%) apparues entre le 2^e^et le 5^e^jour en post réinjection des CSP, et les mucites ont été notées chez 33 patients (51,5%) tous grades confondus. Un épisode fébrile documenté ou non a été noté chez 46 patients (71,8%).

### Evolution

**Figure 1:**
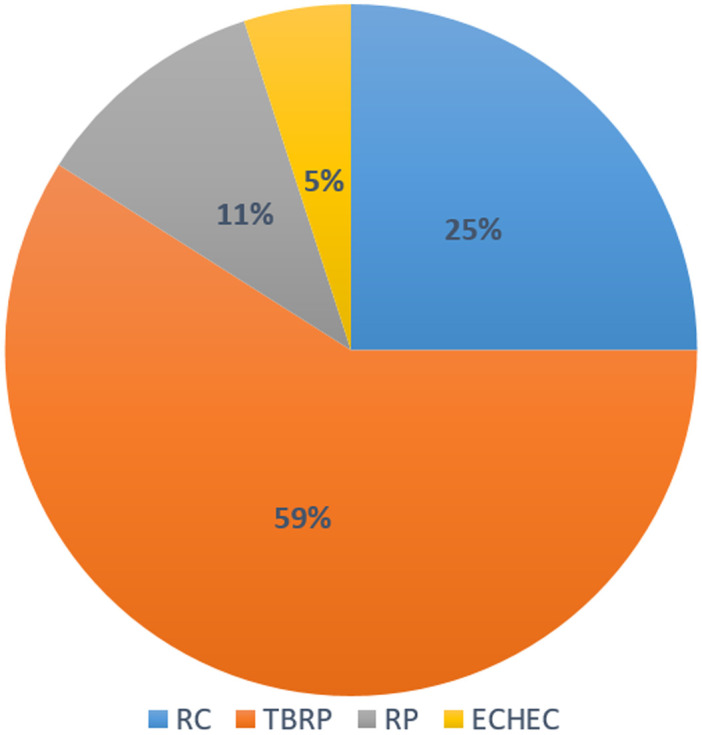
évaluation de la réponse thérapeutique des patients suivis pour myélome multiple à J100 en post-autogreffe des cellules souches hématopoïétiques

**Figure 2:**
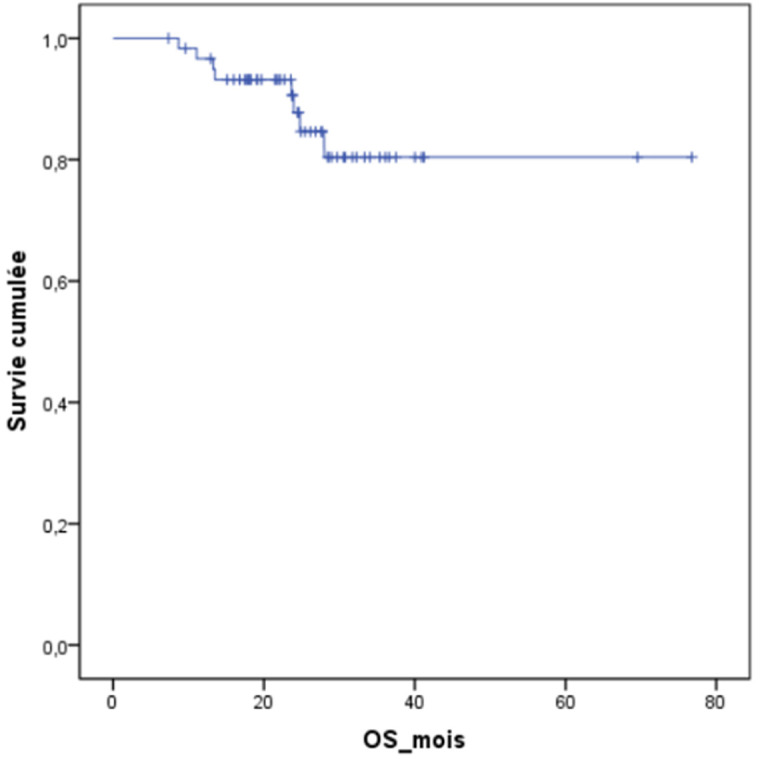
survie globale estimée des patients suivis pour myélome multiple ayant bénéficié d´une autogreffe des cellules souches hématopoïétiques sans cryoconservation

**Figure 3:**
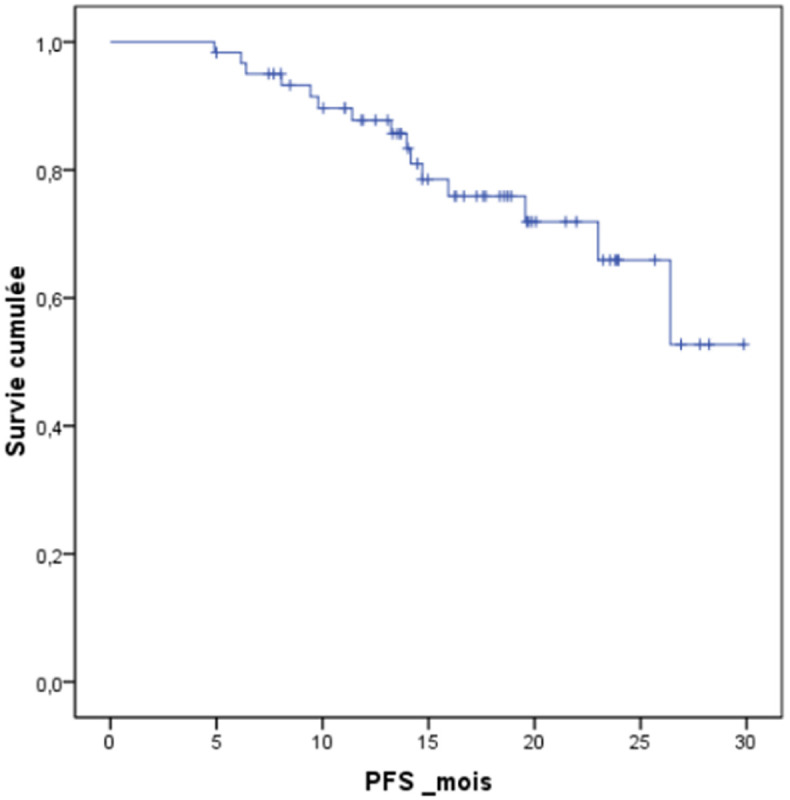
survie sans progression des patients suivis pour myélome multiple ayant bénéficié d´une autogreffe des cellules souches hématopoïétiques sans cryoconservation

Sur 61 patients vivants en post ASCT, l´évaluation réalisée à J100 a montré une réponse globale (RC+TBRP) de 84% (51/61) (Figure 1). La chimiothérapie de consolidation a été reçue dans 47,5% (29 patients), dont 62% (18 patients) ont reçu des cures VTD, et 38% (11 patients) des cures CTD. La mortalité liée au traitement (TRM) à 100 jours d´ASCT était notée dans 3 cas soit 4,68% dont deux sont décédés dans un tableau de choc septique et un patient dans le cadre d´une détresse respiratoire secondaire à une embolie pulmonaire. A 24 mois, l´OS estimée était à 83,5% (ES, 0,05), et l´OS médiane n´a pas été atteinte (Figure 2). A 24 mois, la PFS estimée était à 65,9% (ES, 0,08), et la PFS médiane était à 24,1 mois avec un IC à 95% [21,7- 26,5 mois] (Figure 3). Quinze patients étaient en rechute, et trois patients étaient en échec. Huit patients sont décédés au cours du suivi dont 5 étaient en rechute, et 3 en échec.

## Discussion

Le traitement standard du MM nouvellement diagnostiqué du sujet jeune jusqu´à l´âge de 65 ans et sans comorbidités reste la chimiothérapie à haute dose suivie d´ASCT [4]. Cette stratégie a été basée sur un certain nombre d´essais randomisés, montrant le bénéfice par rapport à la chimiothérapie conventionnelle en termes de PFS [7] et dans certains essais en terme d´OS [3,4]. La perspective d´une qualité de vie meilleure sans symptômes et sans toxicité de la thérapie reste l´élément le plus convaincant de cette approche [3,4,11]. D´après les données précédemment rapportées avec la chimiothérapie à haute dose suivie d´ASCT, le taux de RC et de TBRP est de 40-50%, la PFS médiane est de 24- 36mois et l´OS médiane est de 5-6 ans [12].

Dans la présente étude, nous avons évalué l´ASCT sans cryoconservation dans le traitement du MM de novo. Cette alternative est adaptée à notre contexte vu le nombre de patients inclus sur la liste nationale d´ASCT avec cryoconservation, les délais d´attente pour obtenir des rendez-vous de prélèvement des CSP et le coût qui reste élevé vu les ressources limitées. D´autre part, l´ASCT sans cryoconservation est adaptée au traitement du MM en raison de la courte durée de vie du Mélphalan qui est le seul médicament utilisé dans la chimiothérapie d´intensification. L´ASCT sans cryoconservation permet donc de réduire le temps et le coût [13].

Dans la présente étude, l´ASCT sans cryoconservation a permis d´obtenir des résultats comparables à l´ASCT avec cryoconservation avec des délais d´attentes moins importantes et des installations moins coûteuses. L´inconvénient principal est de ne pas se disposer de CSP de sécurité en cas d´échec de greffe, même si cela n´a pas eu lieu chez aucun de nos patients, ou d´un greffon de réserve pour une 2^e^ autogreffe vue la difficulté de mobilisation des cellules souches après un traitement par le Mélphalan.

L´âge médian dans notre série était à 57 ans [30-65 ans] dont 21,8% étaient âgés ≤ 50 ans. Une population qui reste jeune et un résultat similaire était retrouvé dans les pays à ressources limitées [14-16]. Un plus de soixante-six (66,7)% des patients avaient un ISS à III, un risque élevé par rapport aux études iranienne et indienne dont les patients ISS à III représentent 18,5% 34,2% respectivement [14,15] et similaire à d´autres séries avec des taux entre 79% et 82% [16,17]; ceci peut être expliqué par le retard de consultation entrainant un retard de diagnostic à temps.

Après un traitement d´induction hétérogène, le taux de réponse (RC+ TBRP) était estimé à 67,2% dans notre série. Ce résultat reste comparable aux résultats des centres utilisant des protocoles hétérogènes avec des taux qui varient entre 34% et 72% [15-17]. Après une mobilisation pendant 4-5 jours par du G-CSF, la richesse moyenne du greffon autologue était de 12,97x10^6^ CD34+/Kg [2,4-53x10^6^ CD34+/Kg] et seulement 3 patients ont nécessité 2 séances de leucaphérèse pour obtenir un greffon riche. Ces résultats semblent être supérieurs à ceux constatés au cours des séries d´ASCT sans cryoconservation avec des taux variant entre 2,9x10^6^ et 7,56x10^6^ CD34+/Kg [14-17]. Ceci peut être expliqué par la technique du recueil des CSP utilisé, et la méthode utilisée pour le comptage des cellules CD34+ du greffon. La durée médiane d´hospitalisation ainsi que le temps de recouvrement hématologique dans notre série étaient similaires aux autres études utilisant l´ASCT sans cryoconservation [14-16]. Il n´a pas été constaté une majoration des toxicités hématologiques et muqueuses et nos résultats sont concordants avec ceux de la littérature [14,17,18].

Dans notre expérience monocentrique de 64 patients, les résultats restent satisfaisants et comparables à ceux retrouvés dans les études représentées dans la littérature et résumées dans le tableau ci-dessous [14-17] (Tableau 2). La réponse globale (RC+TBRP) dans notre étude a atteint 84% en post-ASCT sans cryoconservation avec une amélioration du taux des patients en RC qui a passé de 3,2% avant ASCT à 25% en post-ASCT; le taux des patients en RP aussi a diminué de 32,8% à 11% au profit de l´obtention d´une TBRP ou une RC en post-ASCT. Ces résultats sont très satisfaisants et similaires à plusieurs études utilisant la même procédure avec des taux de réponse globale allant de 88-100% [14-16]. La TRM à 100 jours d´ASCT dans notre étude a atteint 4,68%, un résultat qui reste comparable aux études utilisant des CSP congelées ou non [14,15,19,20].

**Tableau 2:** taux et médianes de durée de la survie globale et de la survie sans progression dans différentes séries utilisant l´autogreffe des cellules souches hématopoïétiques sans cryoconservation dans le traitement du myélome multiple

Auteur/Référence	Pays	Série (N)	Durée d´étude	Suivi médian (mois)	OS médiane	PFS médiane	OS (%) 24 mois	PFS (%) 24 mois
Kayal S. *et al*. [15]	Inde	92	18 ans	38,8	61,7	35,4	85	63
Ramzi M. *et al*. [14]	Iran	38	6 ans	31	30	27	76,3	62
Lopez-Otero A. *et al*. [17]	Mexique	26	14 ans	-	NA	34	80	-
Bekadja MA. *et al*. [16]	Algérie	54	26 mois	-	NA	_	93,8	-
Série SHOP	Maroc	64	24 mois	25	NA	24.1	83,5	65,9

OS: survie globale, PFS: survie sans progression, SHOP: service d´Hématologie et Oncologie Pédiatrique

## Conclusion

Malgré le délai court du suivi, les résultats de cette étude sont encourageants et globalement satisfaisants et méritent d´être repris dans une étude plus exhaustive afin de stratifier les groupes de patients et d´affiner les facteurs de risque pour une prise en charge plus adaptée et plus adéquate.

### Etat des connaissances sur le sujet

- La chimiothérapie haute dose suivie d´ASCT reste le traitement de référence du myélome multiple de novo du sujet jeune ≤ 65 ans;
- La cryoconservation ces cellules souches est normalement utilisée dans la procédure d´ASCT;
- L´ASCT sans cryoconservation est une alternative pour les pays à ressources limitées.

### Contribution de notre étude à la connaissance

- L´autogreffe des cellules souches hématopoïétiques sans cryoconservation est faisable et sure dans le traitement du myélome multiple de novo;
- Les résultats de l´autogreffe des cellules souches hématopoïétiques avec cryoconservation sont comparables à ceux de l´autogreffe avec cryoconservation;
- L´autogreffe des cellules souches hématopoïétiques avec cryoconservation permet de réduire les délais d´attente des patients éligibles et d´économiser le coût de la procédure et les installations nécessaires à la cryoconservation.
